# Poly[[μ_4_-tartrato-cadmium(II)] 0.167-hydrate]

**DOI:** 10.1107/S1600536809016882

**Published:** 2009-05-14

**Authors:** Li-Zhen Zhao, Ping Li, Bao-Liang Cao, Seik Weng Ng

**Affiliations:** a Department of Chemistry, Jining Normal College, Wulanchabu, Inner Mongolia 012000, People’s Republic of China; b Department of Chemistry, University of Malaya, 50603 Kuala Lumpur, Malaysia

## Abstract

The title compound, {[Cd(C_4_H_4_O_6_)]·0.167H_2_O}_*n*_, adopts a three-dimensional network structure in which each Cd^II^ ion is chelated by two pairs of carboxyl­ate and hydroxyl O atoms from two tartrate anions, and is additionally linked to two O atoms of two carboxyl­ate groups that are not involved in chelation. The asymmetric unit has four independent cadmium atoms, two of which lie on special positions of 2 site symmetry. The tartrate anions all lie on general positions. All hydroxyl groups are engaged in O—H⋯O hydrogen-bonds, one of which is also bifurcated. The non-coordinating water molecule is situated on a site with half-occupation.

## Related literature

For the structure of cadmium tartrate trihydrate, see: González-Silgo *et al.* (1999 ▶).
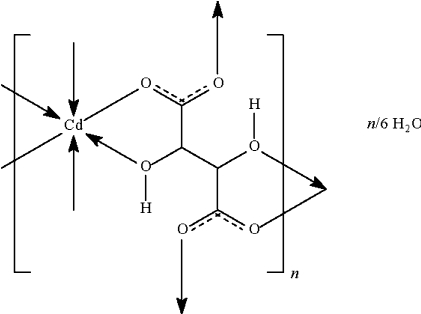


## Experimental

### 

#### Crystal data

[Cd(C_4_H_4_O_6_)]·0.167H_2_O
*M* *_r_* = 263.47
Orthorhombic, 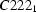

*a* = 10.7901 (4) Å
*b* = 11.1995 (5) Å
*c* = 30.588 (1) Å
*V* = 3696.3 (3) Å^3^
*Z* = 24
Mo *K*α radiation
μ = 3.53 mm^−1^
*T* = 293 K
0.37 × 0.22 × 0.15 mm

#### Data collection

Bruker APEXII area-detector difractometer diffractometer
Absorption correction: multi-scan (*SADABS*, Sheldrick, 1996 ▶) *T*_min_ = 0.505, *T*_max_ = 0.780 (expected range = 0.382–0.589)
13041 measured reflections
4095 independent reflections
4073 reflections with *I* > 2σ(*I*)
*R*_int_ = 0.018

#### Refinement

*R*[*F* ^2^ > 2σ(*F* ^2^)] = 0.022
*wR*(*F* ^2^) = 0.060
*S* = 1.02
4095 reflections
308 parameters
6 restraints
H-atom parameters constrained
Δρ_max_ = 1.34 e Å^−3^
Δρ_min_ = −1.18 e Å^−3^
Absolute structure: Flack (1983 ▶), 1733 Friedel pairs
Flack parameter: −0.02 (2)

### 

Data collection: *APEX2* (Bruker, 2007 ▶); cell refinement: *SAINT* (Bruker, 2007 ▶); data reduction: *SAINT*; program(s) used to solve structure: *SHELXS97* (Sheldrick, 2008 ▶); program(s) used to refine structure: *SHELXL97* (Sheldrick, 2008 ▶); molecular graphics: *X-SEED* (Barbour, 2001 ▶); software used to prepare material for publication: *publCIF* (Westrip, 2009 ▶).

## Supplementary Material

Crystal structure: contains datablocks global, I. DOI: 10.1107/S1600536809016882/ci2796sup1.cif

Structure factors: contains datablocks I. DOI: 10.1107/S1600536809016882/ci2796Isup2.hkl

Additional supplementary materials:  crystallographic information; 3D view; checkCIF report

## Figures and Tables

**Table 1:** Hydrogen-bond geometry (Å, °)

*D*—H⋯*A*	*D*—H	H⋯*A*	*D*⋯*A*	*D*—H⋯*A*
O3—H3⋯O18^i^	0.82	1.96	2.740 (4)	159
O4—H4⋯O10^ii^	0.82	2.50	3.236 (6)	149
O9—H9⋯O11^iii^	0.82	2.17	2.797 (5)	134
O9—H9⋯O1w	0.82	2.12	2.68 (2)	125
O10—H10⋯O15^ii^	0.82	2.15	2.938 (4)	160
O15—H15⋯O1^iv^	0.82	2.13	2.717 (4)	128
O16—H16⋯O7^v^	0.82	1.84	2.609 (4)	155
O1*W*—H1*W*1⋯O14^iii^	0.82	2.26	3.034 (19)	157

Symmetry codes: (i) 
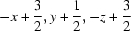
; (ii) 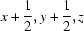; (iii) 
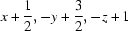
; (iv) 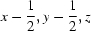; (v) 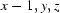.

**Fig. 1.**
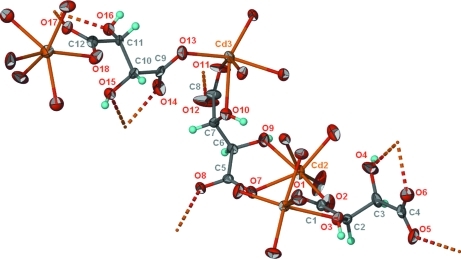
Displacement ellipsoid plot (Barbour, 2001) of a portion of polymeric cadmium tartrate 1/6 hydrate at the 70% probability level; H atoms are drawn as spheres of arbitrary radius. The disordered water molecule is not shown.

## supplementary crystallographic information

### Experimental

Cadmium chloride 2.5 hydrate (0.23 g), *R*,*R*-tartaric acid (0.48 g), sodium hydroxide (0.39 g), imidazole (0.12 g) and water (0.4 ml) were sealed in a 25-ml Teflon-lined stainless-steel vessel. This was heated at 393 K for 3 d. The colourless crystals found in the cooled vessel were picked out manually.

### Refinement

C-bound H-atoms were placed in calculated positions (C-H = 0.98 Å) and were included in the refinement in the riding model approximation, with *U*(H) fixed at 1.2*U*(C). The hydroxy H-atoms were generated geometrically by assuming an *sp*^3^ type of hybridization (O-H = 0.82 Å); these were included in the refinement. At this stage, the difference Fourier map had a peak at about 1.5 Å from hydroxyl H9 atom and it was refined as a water molecule of half-site occupancy as the peak was 2.5 Å from the symmetry-related atom. The two water H atoms were placed in chemically sensible positions; only one of these H atom forms a hydrogen bond to an acceptor oxygen atom. The final difference Fourier map had a peak at 2 Å from O1W and hole at 1.2 Å from Cd1.

### Crystal data

**Table:**

[Cd(C_4_H_4_O_6_)]·0.167H_2_O	*F*(000) = 3016
*M**_r_* = 263.47	*D*_x_ = 2.841 Mg m^−^^3^
Orthorhombic, *C*222_1_	Mo *K*α radiation, λ = 0.71073 Å
Hall symbol: C 2c 2	Cell parameters from 4135 reflections
*a* = 10.7901 (4) Å	θ = 2.3–27.2°
*b* = 11.1995 (5) Å	µ = 3.53 mm^−^^1^
*c* = 30.588 (1) Å	*T* = 293 K
*V* = 3696.3 (3) Å^3^	Block, colourless
*Z* = 24	0.37 × 0.22 × 0.15 mm

### Data collection

**Table:**

Bruker APEXII area-detector difractometer diffractometer	4095 independent reflections
Radiation source: fine-focus sealed tube	4073 reflections with *I* > 2σ(*I*)
graphite	*R*_int_ = 0.018
φ and ω scans	θ_max_ = 27.5°, θ_min_ = 2.7°
Absorption correction: multi-scan (*SADABS*, Sheldrick, 1996)	*h* = −13→12
*T*_min_ = 0.505, *T*_max_ = 0.780	*k* = −14→14
13041 measured reflections	*l* = −38→39

### Refinement

**Table:**

Refinement on *F*^2^	Secondary atom site location: difference Fourier map
Least-squares matrix: full	Hydrogen site location: inferred from neighbouring sites
*R*[*F*^2^ > 2σ(*F*^2^)] = 0.022	H-atom parameters constrained
*wR*(*F*^2^) = 0.060	*w* = 1/[σ^2^(*F*_o_^2^) + (0.0411*P*)^2^ + 9.1485*P*] where *P* = (*F*_o_^2^ + 2*F*_c_^2^)/3
*S* = 1.02	(Δ/σ)_max_ = 0.001
4095 reflections	Δρ_max_ = 1.34 e Å^−^^3^
308 parameters	Δρ_min_ = −1.18 e Å^−^^3^
6 restraints	Absolute structure: Flack (1983), 1733 Friedel pairs
Primary atom site location: structure-invariant direct methods	Flack parameter: −0.02 (2)

### Fractional atomic coordinates and isotropic or equivalent isotropic displacement parameters (Å^2^)

**Table:**

	*x*	*y*	*z*	*U*_iso_*/*U*_eq_	Occ. (<1)
Cd1	1.0000	0.70141 (4)	0.7500	0.01735 (8)	
Cd2	0.95531 (3)	0.81768 (3)	0.578406 (9)	0.02161 (7)	
Cd3	0.41091 (3)	0.79800 (2)	0.601933 (8)	0.01865 (7)	
Cd4	0.0000	0.19902 (4)	0.7500	0.03003 (11)	
O1	1.0079 (3)	0.7619 (3)	0.67881 (10)	0.0233 (6)	
O2	1.1079 (3)	0.8548 (3)	0.62512 (10)	0.0302 (7)	
O3	1.1495 (3)	0.8566 (3)	0.74078 (9)	0.0208 (6)	
H3	1.2130	0.8367	0.7536	0.031*	
O4	1.0508 (4)	1.0602 (3)	0.69188 (15)	0.0482 (11)	
H4	1.0337	1.0916	0.6684	0.072*	
O5	1.3661 (3)	1.0768 (3)	0.72008 (12)	0.0317 (7)	
O6	1.1993 (3)	1.1322 (3)	0.75792 (11)	0.0293 (7)	
O7	0.9280 (3)	0.6190 (3)	0.58705 (11)	0.0295 (7)	
O8	0.8178 (3)	0.4562 (3)	0.57088 (11)	0.0253 (6)	
O9	0.7827 (3)	0.7584 (3)	0.53809 (12)	0.0310 (7)	
H9	0.7944	0.7771	0.5125	0.047*	
O10	0.6037 (3)	0.6797 (2)	0.59740 (9)	0.0195 (5)	
H10	0.6630	0.7235	0.6023	0.029*	
O11	0.4479 (3)	0.7476 (3)	0.53234 (10)	0.0320 (7)	
O12	0.5479 (4)	0.6380 (4)	0.48342 (11)	0.0463 (10)	
O13	0.2972 (3)	0.6471 (3)	0.62762 (11)	0.0276 (7)	
O14	0.3822 (3)	0.5056 (3)	0.58715 (11)	0.0327 (8)	
O15	0.2955 (3)	0.3293 (3)	0.63798 (9)	0.0199 (5)	
H15	0.3205	0.2918	0.6592	0.030*	
O16	0.0760 (3)	0.4462 (2)	0.61037 (9)	0.0213 (6)	
H16	0.0493	0.5109	0.6021	0.032*	
O17	−0.0251 (2)	0.3105 (3)	0.67293 (9)	0.0217 (5)	
O18	0.1216 (3)	0.3518 (3)	0.72046 (10)	0.0273 (7)	
O1W	0.6901 (17)	0.9073 (17)	0.4779 (6)	0.145 (7)	0.50
H1W1	0.7356	0.9133	0.4565	0.218*	0.50
H1W2	0.6878	0.9711	0.4910	0.218*	0.50
C1	1.0921 (4)	0.8279 (3)	0.66460 (12)	0.0178 (7)	
C2	1.1821 (3)	0.8874 (3)	0.69695 (12)	0.0160 (7)	
H2C	1.2666	0.8599	0.6909	0.019*	
C3	1.1755 (4)	1.0224 (4)	0.69036 (15)	0.0257 (9)	
H3C	1.2103	1.0423	0.6617	0.031*	
C4	1.2533 (4)	1.0834 (3)	0.72602 (13)	0.0220 (8)	
C5	0.8399 (4)	0.5650 (4)	0.56782 (13)	0.0168 (7)	
C6	0.7517 (4)	0.6364 (3)	0.53935 (12)	0.0174 (7)	
H6C	0.7558	0.6047	0.5095	0.021*	
C7	0.6197 (3)	0.6243 (3)	0.55551 (12)	0.0160 (7)	
H7C	0.6016	0.5390	0.5588	0.019*	
C8	0.5297 (4)	0.6755 (4)	0.52122 (13)	0.0234 (8)	
C9	0.3210 (4)	0.5408 (4)	0.61868 (13)	0.0191 (8)	
C10	0.2672 (3)	0.4479 (3)	0.65126 (12)	0.0155 (7)	
H10C	0.3020	0.4622	0.6804	0.019*	
C11	0.1258 (4)	0.4612 (3)	0.65322 (13)	0.0168 (7)	
H11C	0.1054	0.5415	0.6638	0.020*	
C12	0.0704 (4)	0.3690 (3)	0.68453 (12)	0.0175 (7)	

### Atomic displacement parameters (Å^2^)

**Table:**

	*U*^11^	*U*^22^	*U*^33^	*U*^12^	*U*^13^	*U*^23^
Cd1	0.01892 (17)	0.01774 (17)	0.01540 (16)	0.000	−0.00203 (12)	0.000
Cd2	0.02609 (15)	0.01939 (14)	0.01935 (13)	−0.00727 (12)	0.00123 (10)	−0.00092 (10)
Cd3	0.02465 (14)	0.01520 (12)	0.01611 (12)	−0.00012 (11)	−0.00171 (9)	0.00103 (11)
Cd4	0.0214 (2)	0.0270 (2)	0.0417 (2)	0.000	0.00595 (16)	0.000
O1	0.0222 (14)	0.0262 (14)	0.0216 (13)	−0.0078 (12)	−0.0076 (11)	0.0021 (11)
O2	0.0414 (19)	0.0331 (17)	0.0163 (13)	−0.0116 (15)	−0.0006 (12)	−0.0019 (12)
O3	0.0218 (14)	0.0251 (14)	0.0156 (13)	−0.0048 (11)	−0.0076 (10)	0.0027 (11)
O4	0.043 (2)	0.0263 (16)	0.075 (3)	0.0144 (16)	−0.039 (2)	−0.0157 (17)
O5	0.0260 (16)	0.0282 (16)	0.0409 (19)	−0.0114 (13)	0.0012 (14)	−0.0064 (15)
O6	0.0278 (16)	0.0292 (15)	0.0308 (17)	−0.0002 (13)	−0.0033 (12)	−0.0115 (13)
O7	0.0274 (17)	0.0166 (12)	0.0447 (19)	0.0003 (12)	−0.0195 (14)	−0.0032 (13)
O8	0.0229 (14)	0.0149 (13)	0.0383 (18)	0.0001 (12)	−0.0082 (12)	0.0011 (12)
O9	0.0288 (18)	0.0210 (14)	0.0432 (19)	−0.0068 (12)	−0.0084 (14)	0.0166 (13)
O10	0.0226 (13)	0.0196 (12)	0.0162 (11)	0.0005 (11)	−0.0020 (10)	−0.0045 (11)
O11	0.0330 (17)	0.0416 (18)	0.0213 (14)	0.0254 (15)	−0.0083 (13)	−0.0069 (13)
O12	0.047 (2)	0.069 (3)	0.0224 (15)	0.038 (2)	−0.0175 (15)	−0.0207 (16)
O13	0.0279 (16)	0.0182 (14)	0.0366 (17)	−0.0058 (12)	0.0073 (13)	0.0002 (13)
O14	0.042 (2)	0.0219 (14)	0.0345 (17)	−0.0030 (14)	0.0219 (15)	0.0005 (13)
O15	0.0222 (14)	0.0177 (13)	0.0200 (13)	0.0019 (11)	−0.0014 (10)	0.0040 (11)
O16	0.0235 (15)	0.0197 (13)	0.0206 (13)	−0.0014 (11)	−0.0066 (11)	0.0055 (10)
O17	0.0200 (14)	0.0240 (14)	0.0211 (12)	−0.0063 (12)	0.0015 (10)	0.0025 (12)
O18	0.0236 (16)	0.0360 (17)	0.0223 (14)	−0.0060 (13)	−0.0009 (11)	0.0037 (13)
O1W	0.130 (10)	0.170 (10)	0.136 (10)	−0.007 (8)	0.010 (8)	0.065 (8)
C1	0.0201 (17)	0.0161 (16)	0.0172 (16)	−0.0026 (15)	−0.0031 (13)	−0.0002 (14)
C2	0.0157 (16)	0.0166 (17)	0.0156 (17)	−0.0012 (14)	0.0003 (13)	−0.0013 (14)
C3	0.036 (2)	0.0153 (18)	0.026 (2)	−0.0032 (16)	−0.0151 (17)	0.0011 (16)
C4	0.028 (2)	0.0134 (16)	0.0244 (19)	−0.0045 (16)	−0.0079 (16)	0.0042 (15)
C5	0.0158 (18)	0.0179 (17)	0.0167 (17)	0.0023 (14)	−0.0004 (12)	−0.0023 (14)
C6	0.0195 (18)	0.0172 (17)	0.0156 (16)	0.0016 (15)	−0.0008 (13)	0.0016 (13)
C7	0.0149 (18)	0.0166 (16)	0.0165 (17)	0.0009 (14)	−0.0041 (13)	−0.0034 (14)
C8	0.023 (2)	0.027 (2)	0.0199 (17)	0.0100 (17)	−0.0087 (14)	−0.0056 (16)
C9	0.0132 (17)	0.0214 (19)	0.0228 (19)	−0.0043 (15)	−0.0052 (14)	−0.0020 (16)
C10	0.0140 (17)	0.0171 (16)	0.0154 (16)	−0.0027 (14)	−0.0022 (13)	0.0002 (13)
C11	0.0162 (18)	0.0117 (15)	0.0224 (19)	−0.0016 (14)	−0.0006 (13)	−0.0008 (14)
C12	0.0182 (19)	0.0187 (16)	0.0157 (16)	−0.0001 (14)	0.0046 (13)	−0.0041 (14)

### Geometric parameters (Å, °)

**Table:**

Cd1—O5^i^	2.207 (3)	O8—Cd3^xi^	2.247 (3)
Cd1—O5^ii^	2.207 (3)	O9—C6	1.408 (5)
Cd1—O1	2.282 (3)	O9—H9	0.82
Cd1—O1^iii^	2.282 (3)	O10—C7	1.434 (4)
Cd1—O3	2.388 (3)	O10—H10	0.82
Cd1—O3^iii^	2.388 (3)	O11—C8	1.245 (5)
Cd2—O12^iv^	2.196 (3)	O12—C8	1.245 (5)
Cd2—O2	2.220 (3)	O12—Cd2^xii^	2.196 (3)
Cd2—O7	2.260 (3)	O13—C9	1.248 (5)
Cd2—O14^v^	2.264 (3)	O14—C9	1.233 (5)
Cd2—O9	2.330 (3)	O14—Cd2^i^	2.264 (3)
Cd2—O15^v^	2.512 (3)	O15—C10	1.422 (5)
Cd3—O13	2.232 (3)	O15—Cd2^i^	2.512 (3)
Cd3—O11	2.238 (3)	O15—H15	0.82
Cd3—O8^vi^	2.247 (3)	O16—C11	1.426 (4)
Cd3—O17^v^	2.283 (3)	O16—Cd3^i^	2.449 (3)
Cd3—O16^v^	2.449 (3)	O16—H16	0.82
Cd3—O10	2.470 (3)	O17—C12	1.272 (5)
Cd4—O6^vii^	2.290 (3)	O17—Cd3^i^	2.283 (3)
Cd4—O6^viii^	2.290 (3)	O18—C12	1.245 (5)
Cd4—O18	2.338 (3)	O1W—H1W1	0.82
Cd4—O18^ix^	2.338 (3)	O1W—H1W2	0.82
Cd4—O4^viii^	2.424 (4)	C1—C2	1.538 (5)
Cd4—O4^vii^	2.424 (4)	C2—C3	1.526 (5)
O1—C1	1.250 (5)	C2—H2C	0.98
O2—C1	1.257 (5)	C3—C4	1.536 (5)
O3—C2	1.428 (5)	C3—H3C	0.98
O3—H3	0.82	C5—C6	1.518 (5)
O4—C3	1.412 (6)	C6—C7	1.514 (5)
O4—Cd4^x^	2.424 (4)	C6—H6C	0.98
O4—H4	0.82	C7—C8	1.540 (5)
O5—C4	1.233 (6)	C7—H7C	0.98
O5—Cd1^v^	2.207 (3)	C9—C10	1.554 (5)
O6—C4	1.261 (5)	C10—C11	1.534 (5)
O6—Cd4^x^	2.290 (3)	C10—H10C	0.98
O7—C5	1.271 (5)	C11—C12	1.530 (5)
O8—C5	1.246 (5)	C11—H11C	0.98
			
O5^i^—Cd1—O5^ii^	101.55 (19)	C6—O9—H9	108.1
O5^i^—Cd1—O1	79.43 (12)	Cd2—O9—H9	108.1
O5^ii^—Cd1—O1	123.99 (12)	C7—O10—Cd3	112.6 (2)
O5^i^—Cd1—O1^iii^	123.99 (12)	C7—O10—H10	109.1
O5^ii^—Cd1—O1^iii^	79.43 (12)	Cd3—O10—H10	109.1
O1—Cd1—O1^iii^	145.46 (15)	C8—O11—Cd3	123.4 (3)
O5^i^—Cd1—O3	148.58 (11)	C8—O12—Cd2^xii^	130.7 (3)
O5^ii^—Cd1—O3	93.84 (12)	C9—O13—Cd3	122.1 (3)
O1—Cd1—O3	69.28 (10)	C9—O14—Cd2^i^	125.2 (3)
O1^iii^—Cd1—O3	85.51 (10)	C10—O15—Cd2^i^	113.8 (2)
O5^i^—Cd1—O3^iii^	93.84 (12)	C10—O15—H15	108.8
O5^ii^—Cd1—O3^iii^	148.58 (11)	Cd2^i^—O15—H15	108.8
O1—Cd1—O3^iii^	85.51 (10)	C11—O16—Cd3^i^	116.8 (2)
O1^iii^—Cd1—O3^iii^	69.28 (9)	C11—O16—H16	108.1
O3—Cd1—O3^iii^	86.61 (15)	Cd3^i^—O16—H16	108.1
O12^iv^—Cd2—O2	100.04 (13)	C12—O17—Cd3^i^	122.8 (2)
O12^iv^—Cd2—O7	112.50 (14)	C12—O18—Cd4	101.8 (3)
O2—Cd2—O7	101.90 (11)	H1W1—O1W—H1W2	109.7
O12^iv^—Cd2—O14^v^	92.86 (14)	O1—C1—O2	125.1 (4)
O2—Cd2—O14^v^	90.47 (13)	O1—C1—C2	119.5 (3)
O7—Cd2—O14^v^	148.89 (13)	O2—C1—C2	115.4 (3)
O12^iv^—Cd2—O9	88.41 (13)	O3—C2—C3	110.6 (3)
O2—Cd2—O9	170.78 (11)	O3—C2—C1	110.1 (3)
O7—Cd2—O9	71.16 (10)	C3—C2—C1	108.3 (3)
O14^v^—Cd2—O9	92.81 (13)	O3—C2—H2C	109.3
O12^iv^—Cd2—O15^v^	157.71 (14)	C3—C2—H2C	109.3
O2—Cd2—O15^v^	91.88 (10)	C1—C2—H2C	109.3
O7—Cd2—O15^v^	82.91 (12)	O4—C3—C2	109.7 (4)
O14^v^—Cd2—O15^v^	68.10 (10)	O4—C3—C4	111.3 (4)
O9—Cd2—O15^v^	81.37 (11)	C2—C3—C4	108.8 (3)
O13—Cd3—O11	104.01 (13)	O4—C3—H3C	109.0
O13—Cd3—O8^vi^	119.99 (12)	C2—C3—H3C	109.0
O11—Cd3—O8^vi^	82.89 (12)	C4—C3—H3C	109.0
O13—Cd3—O17^v^	82.97 (11)	O5—C4—O6	126.6 (4)
O11—Cd3—O17^v^	150.04 (10)	O5—C4—C3	114.1 (4)
O8^vi^—Cd3—O17^v^	119.27 (11)	O6—C4—C3	119.4 (4)
O13—Cd3—O16^v^	151.21 (11)	O8—C5—O7	125.0 (4)
O11—Cd3—O16^v^	98.13 (12)	O8—C5—C6	116.0 (3)
O8^vi^—Cd3—O16^v^	80.54 (10)	O7—C5—C6	119.0 (4)
O17^v^—Cd3—O16^v^	68.79 (10)	O9—C6—C7	108.6 (3)
O13—Cd3—O10	94.40 (11)	O9—C6—C5	112.2 (3)
O11—Cd3—O10	70.20 (10)	C7—C6—C5	110.8 (3)
O8^vi^—Cd3—O10	140.87 (11)	O9—C6—H6C	108.4
O17^v^—Cd3—O10	80.31 (9)	C7—C6—H6C	108.4
O16^v^—Cd3—O10	75.93 (9)	C5—C6—H6C	108.4
O6^vii^—Cd4—O6^viii^	141.86 (17)	O10—C7—C6	111.5 (3)
O6^vii^—Cd4—O18	136.50 (11)	O10—C7—C8	111.8 (3)
O6^viii^—Cd4—O18	75.69 (12)	C6—C7—C8	109.7 (3)
O6^vii^—Cd4—O18^ix^	75.69 (12)	O10—C7—H7C	107.9
O6^viii^—Cd4—O18^ix^	136.50 (11)	C6—C7—H7C	107.9
O18—Cd4—O18^ix^	85.92 (17)	C8—C7—H7C	107.9
O6^vii^—Cd4—O4^viii^	85.73 (13)	O11—C8—O12	125.8 (4)
O6^viii^—Cd4—O4^viii^	69.85 (11)	O11—C8—C7	120.2 (3)
O18—Cd4—O4^viii^	93.37 (14)	O12—C8—C7	114.1 (4)
O18^ix^—Cd4—O4^viii^	151.60 (11)	O14—C9—O13	125.9 (4)
O6^vii^—Cd4—O4^vii^	69.85 (11)	O14—C9—C10	119.2 (4)
O6^viii^—Cd4—O4^vii^	85.73 (13)	O13—C9—C10	114.9 (4)
O18—Cd4—O4^vii^	151.60 (11)	O15—C10—C11	108.4 (3)
O18^ix^—Cd4—O4^vii^	93.37 (14)	O15—C10—C9	111.2 (3)
O4^viii^—Cd4—O4^vii^	100.2 (2)	C11—C10—C9	109.4 (3)
C1—O1—Cd1	122.3 (2)	O15—C10—H10C	109.3
C1—O2—Cd2	118.2 (3)	C11—C10—H10C	109.3
C2—O3—Cd1	116.9 (2)	C9—C10—H10C	109.3
C2—O3—H3	108.1	O16—C11—C12	110.4 (3)
Cd1—O3—H3	108.1	O16—C11—C10	109.1 (3)
C3—O4—Cd4^x^	115.6 (3)	C12—C11—C10	110.3 (3)
C3—O4—H4	108.4	O16—C11—H11C	109.0
Cd4^x^—O4—H4	108.4	C12—C11—H11C	109.0
C4—O5—Cd1^v^	123.2 (3)	C10—C11—H11C	109.0
C4—O6—Cd4^x^	119.6 (3)	O18—C12—O17	121.7 (4)
C5—O7—Cd2	120.8 (3)	O18—C12—C11	118.9 (3)
C5—O8—Cd3^xi^	135.9 (3)	O17—C12—C11	119.3 (3)
C6—O9—Cd2	116.9 (2)		
			
O5^i^—Cd1—O1—C1	−165.4 (3)	C1—C2—C3—C4	173.4 (3)
O5^ii^—Cd1—O1—C1	−68.6 (3)	Cd1^v^—O5—C4—O6	−23.0 (6)
O1^iii^—Cd1—O1—C1	57.5 (3)	Cd1^v^—O5—C4—C3	158.2 (3)
O3—Cd1—O1—C1	11.7 (3)	Cd4^x^—O6—C4—O5	155.8 (3)
O3^iii^—Cd1—O1—C1	99.8 (3)	Cd4^x^—O6—C4—C3	−25.5 (5)
O12^iv^—Cd2—O2—C1	−173.2 (3)	O4—C3—C4—O5	−163.6 (4)
O7—Cd2—O2—C1	−57.5 (3)	C2—C3—C4—O5	75.5 (5)
O14^v^—Cd2—O2—C1	93.8 (3)	O4—C3—C4—O6	17.6 (5)
O15^v^—Cd2—O2—C1	25.7 (3)	C2—C3—C4—O6	−103.4 (4)
O5^i^—Cd1—O3—C2	−6.4 (4)	Cd3^xi^—O8—C5—O7	−6.2 (7)
O5^ii^—Cd1—O3—C2	113.2 (3)	Cd3^xi^—O8—C5—C6	175.0 (3)
O1—Cd1—O3—C2	−11.8 (2)	Cd2—O7—C5—O8	−179.7 (3)
O1^iii^—Cd1—O3—C2	−167.7 (3)	Cd2—O7—C5—C6	−0.9 (5)
O3^iii^—Cd1—O3—C2	−98.3 (3)	Cd2—O9—C6—C7	123.0 (3)
O12^iv^—Cd2—O7—C5	−79.3 (4)	Cd2—O9—C6—C5	0.1 (4)
O2—Cd2—O7—C5	174.4 (3)	O8—C5—C6—O9	179.4 (4)
O14^v^—Cd2—O7—C5	63.0 (4)	O7—C5—C6—O9	0.5 (5)
O9—Cd2—O7—C5	0.7 (3)	O8—C5—C6—C7	57.8 (5)
O15^v^—Cd2—O7—C5	83.9 (3)	O7—C5—C6—C7	−121.1 (4)
O12^iv^—Cd2—O9—C6	114.1 (3)	Cd3—O10—C7—C6	136.4 (2)
O7—Cd2—O9—C6	−0.4 (3)	Cd3—O10—C7—C8	13.1 (4)
O14^v^—Cd2—O9—C6	−153.1 (3)	O9—C6—C7—O10	−56.7 (4)
O15^v^—Cd2—O9—C6	−85.8 (3)	C5—C6—C7—O10	67.0 (4)
O13—Cd3—O10—C7	91.5 (2)	O9—C6—C7—C8	67.8 (4)
O11—Cd3—O10—C7	−11.8 (2)	C5—C6—C7—C8	−168.5 (3)
O8^vi^—Cd3—O10—C7	−61.3 (3)	Cd3—O11—C8—O12	174.4 (4)
O17^v^—Cd3—O10—C7	173.6 (2)	Cd3—O11—C8—C7	−5.6 (6)
O16^v^—Cd3—O10—C7	−116.0 (2)	Cd2^xii^—O12—C8—O11	2.3 (9)
O13—Cd3—O11—C8	−80.2 (4)	Cd2^xii^—O12—C8—C7	−177.7 (3)
O8^vi^—Cd3—O11—C8	160.6 (4)	O10—C7—C8—O11	−6.3 (6)
O17^v^—Cd3—O11—C8	20.2 (5)	C6—C7—C8—O11	−130.6 (4)
O16^v^—Cd3—O11—C8	81.3 (4)	O10—C7—C8—O12	173.7 (4)
O10—Cd3—O11—C8	9.5 (4)	C6—C7—C8—O12	49.5 (5)
O11—Cd3—O13—C9	41.6 (4)	Cd2^i^—O14—C9—O13	167.6 (3)
O8^vi^—Cd3—O13—C9	131.4 (3)	Cd2^i^—O14—C9—C10	−12.1 (5)
O17^v^—Cd3—O13—C9	−108.7 (3)	Cd3—O13—C9—O14	−21.2 (6)
O16^v^—Cd3—O13—C9	−97.5 (4)	Cd3—O13—C9—C10	158.5 (2)
O10—Cd3—O13—C9	−29.0 (3)	Cd2^i^—O15—C10—C11	132.7 (2)
O6^vii^—Cd4—O18—C12	−25.4 (3)	Cd2^i^—O15—C10—C9	12.4 (3)
O6^viii^—Cd4—O18—C12	130.2 (3)	O14—C9—C10—O15	−1.9 (5)
O18^ix^—Cd4—O18—C12	−89.6 (3)	O13—C9—C10—O15	178.3 (3)
O4^viii^—Cd4—O18—C12	61.9 (3)	O14—C9—C10—C11	−121.7 (4)
O4^vii^—Cd4—O18—C12	−179.1 (3)	O13—C9—C10—C11	58.6 (4)
Cd1—O1—C1—O2	172.9 (3)	Cd3^i^—O16—C11—C12	5.5 (4)
Cd1—O1—C1—C2	−10.0 (5)	Cd3^i^—O16—C11—C10	126.9 (3)
Cd2—O2—C1—O1	14.3 (6)	O15—C10—C11—O16	−63.9 (4)
Cd2—O2—C1—C2	−162.9 (2)	C9—C10—C11—O16	57.6 (4)
Cd1—O3—C2—C3	130.8 (3)	O15—C10—C11—C12	57.6 (4)
Cd1—O3—C2—C1	11.1 (4)	C9—C10—C11—C12	179.0 (3)
O1—C1—C2—O3	−1.4 (5)	Cd4—O18—C12—O17	1.1 (4)
O2—C1—C2—O3	176.0 (3)	Cd4—O18—C12—C11	−177.6 (3)
O1—C1—C2—C3	−122.5 (4)	Cd3^i^—O17—C12—O18	−161.8 (3)
O2—C1—C2—C3	54.9 (5)	Cd3^i^—O17—C12—C11	16.8 (5)
Cd4^x^—O4—C3—C2	118.3 (3)	O16—C11—C12—O18	164.7 (3)
Cd4^x^—O4—C3—C4	−2.1 (5)	C10—C11—C12—O18	44.1 (5)
O3—C2—C3—O4	−69.3 (4)	O16—C11—C12—O17	−14.0 (5)
C1—C2—C3—O4	51.5 (5)	C10—C11—C12—O17	−134.7 (4)
O3—C2—C3—C4	52.7 (5)		

Symmetry codes: (i) *x*−1/2, *y*−1/2, *z*; (ii) −*x*+5/2, *y*−1/2, −*z*+3/2; (iii) −*x*+2, *y*, −*z*+3/2; (iv) *x*+1/2, −*y*+3/2, −*z*+1; (v) *x*+1/2, *y*+1/2, *z*; (vi) *x*−1/2, *y*+1/2, *z*; (vii) −*x*+1, *y*−1, −*z*+3/2; (viii) *x*−1, *y*−1, *z*; (ix) −*x*, *y*, −*z*+3/2; (x) *x*+1, *y*+1, *z*; (xi) *x*+1/2, *y*−1/2, *z*; (xii) *x*−1/2, −*y*+3/2, −*z*+1.

### Hydrogen-bond geometry (Å, °)

**Table:**

*D*—H···*A*	*D*—H	H···*A*	*D*···*A*	*D*—H···*A*
O3—H3···O18^xiii^	0.82	1.96	2.740 (4)	159
O4—H4···O10^v^	0.82	2.50	3.236 (6)	149
O9—H9···O11^iv^	0.82	2.17	2.797 (5)	134
O9—H9···O1w	0.82	2.12	2.68 (2)	125
O10—H10···O15^v^	0.82	2.15	2.938 (4)	160
O15—H15···O1^i^	0.82	2.13	2.717 (4)	128
O16—H16···O7^xiv^	0.82	1.84	2.609 (4)	155
O1W—H1W1···O14^iv^	0.82	2.26	3.034 (19)	157

Symmetry codes: (xiii) −*x*+3/2, *y*+1/2, −*z*+3/2; (v) *x*+1/2, *y*+1/2, *z*; (iv) *x*+1/2, −*y*+3/2, −*z*+1; (i) *x*−1/2, *y*−1/2, *z*; (xiv) *x*−1, *y*, *z*.
